# Glandular and Non-Glandular Trichomes from *Phlomis herba-venti* subsp. *pungens* Leaves: Light, Confocal, and Scanning Electron Microscopy and Histochemistry of the Secretory Products

**DOI:** 10.3390/plants12132423

**Published:** 2023-06-23

**Authors:** Irina Neta Gostin

**Affiliations:** Faculty of Biology, “Alexandru Ioan Cuza” University of Iași, Bdul Carol I, no. 11, 700506 Iasi, Romania; irinagostin@yahoo.com; Tel.: +402-3220-1510

**Keywords:** trichomes, *Phlomis*, microscopy, SEM, histochemistry

## Abstract

The purpose of this paper is to highlight the morphological peculiarities of glandular and non-glandular trichomes from leaves of *Phlomis herba-venti* subsp. *Pungens* using light, confocal, and scanning electron microscopy. Histochemistry techniques were used to analyze the localization of different chemical compounds in secretory trichomes. Two types of non-glandular trichomes were identified: unicellular and branched. They were found more frequently on the lower epidermis of leaves in different stages of ontogenetic development. Glandular trichomes were categorized as capitate (C1 and C2) with different stalk lengths and one–four secretory cells and dendroids (D) with one–four secretory cells. The histochemical analyses revealed distinct secretory products in terms of composition and distribution among the three types of glandular trichomes. The dendroid category of glandular trichomes is rarely found in plants and is not characteristic of the Lamiaceae species. They were described and characterized from a micromorphological and histochemical point of view for the first time in *P. herba-venti*.

## 1. Introduction

Plant hairs, or trichomes, serve a variety of defensive and physiological functions [1]. Non-glandular trichomes play a crucial role in plant defense by reducing transpiration, increasing tolerance to freezing, and deflecting intense solar radiation, thereby reducing herbivory [2,3]. Glandular trichomes, through the volatile oils they secrete, act as a barrier against various external factors such as herbivores, pathogens, UV-B radiation, extreme temperatures, and drought [4]; they may also attract pollinators [5,6]. Additionally, different trichome types have significant systematic value [7].

The genus *Phlomis* L. contains over 100 species [8], distributed throughout Europe, Africa, and Asia. Many of them have aromatic and medicinal characteristics [9]. *Phlomis herba-venti* subsp. *pungens* (Willd.) Maire ex DeFillips (synonym with *Phlomis pungens* Willd [10]), is a species with a history of use in traditional medicine as a stimulant, tonic, and diuretic. It also exhibits antimicrobial activity, particularly against Gram-positive microorganisms [11], as well as anti-inflammatory properties [12]. 

There are many structural, ultrastructural, histochemical, and chemical studies regarding glandular trichomes from some Lamiaceae species [13,14,15,16,17,18,19,20,21,22,23,24].The trichomes from the leaves of *Phlomis fruticosa* were investigated by Nikolakaki and Christodoulakis [25]. Similar studies have also been conducted on *P. olivieri* [26], *P. russeliana* [16], and *P. monocephala* [27]. No information is available on the structural, histochemical, and micromorphological aspects of trichomes of *Phlomis herba-venti*. Despite the lack of studies on the morphology of secretory trichomes from this species, numerous investigations have been carried out on the composition of the volatile oils produced by them [28,29,30,31,32]. The major constituents of the volatile oil extracted from the leaves of this plant are germacrene D, hexadecanoic acid, and α-pinene [32].

The aim of this paper was to study, using light, confocal, and scanning electron microscopy, the morphology and secretory products of glandular trichomes of *Phlomis herba-venti* subsp. *pungens*. The medicinal uses of this species make a better understanding of the secretory structures necessary. Furthermore, through histochemistry studies, the composition of secretion products can be more accurately correlated with the morphology of glandular trichomes, which are described in detail in this paper for the first time.

## 2. Results

**Figure 1:**
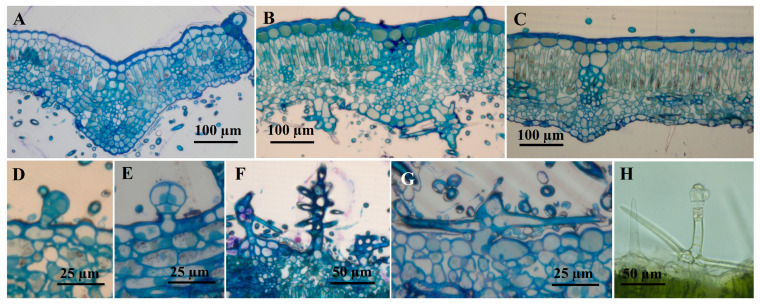
Cross sections through the leaf of *Phlomis verba-venti*. (**A**–**G**) Sections through plant material included in epoxy resin E812 and stained with toluidine blue; (**H**) freehand section made on fresh material, uncolored). (**A**) very young leaf; (**B**) intermediate leaf; (**C**) fully expanded leaf; (**D**,**E**) capitate glandular trichomes (C1), lower epidermis, intermediate leaf; (**F**,**G**) non-glandular dendroid trichomes, lower epidermis, intermediate leaf; (**H**) glandular trichome dendroid, upper epidermis, intermediate leaf.

**Figure 2:**
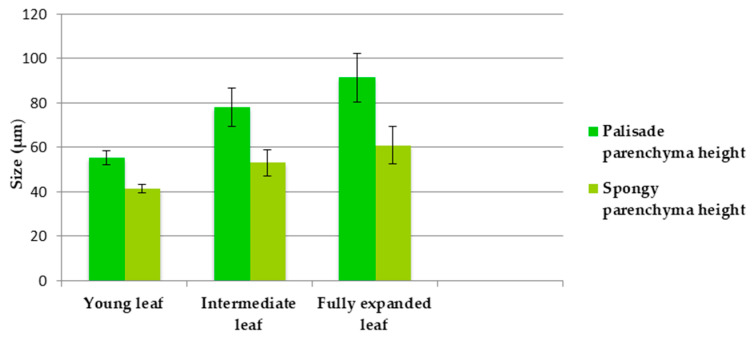
The sizes of palisadic parenchyma and spongy parenchyma in leaves of different ages (average ± standard error).

In cross-section, the *P. herba venti* leaf is dorsiventral bifacial, with well-developed palisade parenchyma under the upper epidermis (usually unilayered, rarely bilayered, with narrow and long cells) (Figure 1). The spongy parenchyma occupies a smaller space than the palisadic one (Figure 2); it consists of small, isodiametric cells, with small air spaces between them. The cells of the upper epidermis are covered by a thick cuticle, which is a xeromorphic character, along with the air-reduced spaces in the leaf mesophyll. 

### 2.1. Trichomes Distribution—Two Types of Trichomes were Observed on the Leaf Epidermis: Glandular (Capitates and Dendroids) and Non-Glandular (Figure 3)

In both cases, a rather pronounced variability of the morphology of the trichomes can be observed. Scanning electron microscopy (SEM) investigations showed an unequal distribution of glandular and non-glandular trichomes on the two sides of the leaf, and this distribution varies with the age of the analyzed leaf.

Table 1 shows the density of glandular and non-glandular trichomes on the upper and lower epidermis in leaves in different stages of development.

**Figure 3:**
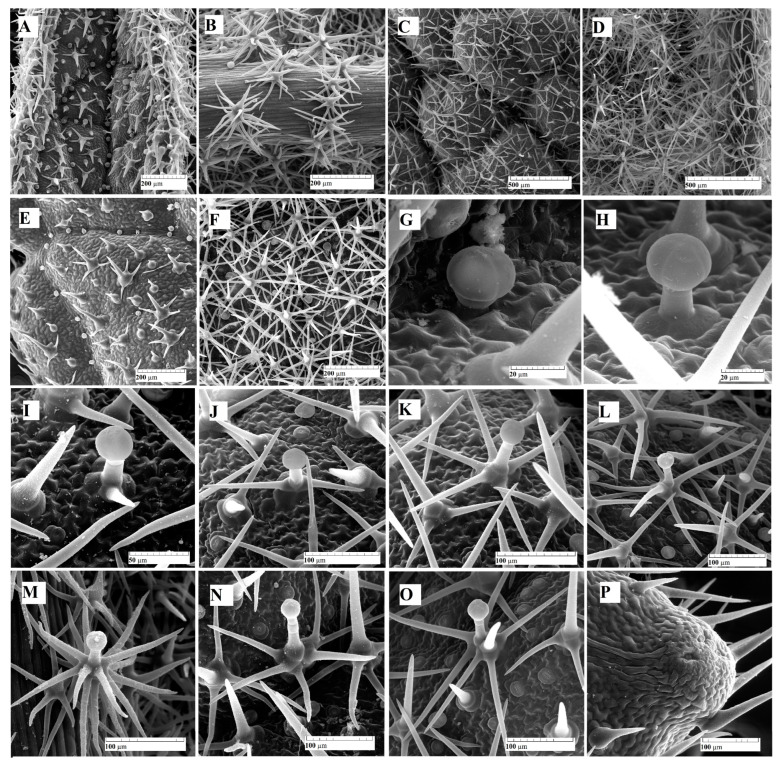
SEM micrographs with glandular and non-glandular trichomes: (**A**) upper epidermis, very young leaf; (**B**) lower epidermis, very young leaf; (**C**) upper epidermis, intermediate leaf; (**D**) lower epidermis, intermediate leaf; (**E**) upper epidermis, fully expanded leaf; (**F**) lower epidermis, fully expanded leaf expanded; (**G**) capitate glandular trichome (C1), upper epidermis, intermediate leaf; (**H**) capitate glandular trichome (C2), upper epidermis, intermediate leaf; (**I**–**L**,**N**,**O**) dendroid glandular trichomes with different degrees of branching (D), upper epidermis, intermediate leaf; (**M**) highly branched glandular dendroid trichome (D), lower epidermis, very young leaf; (**P**) hydathode on the edge of the mature leaf, upper epidermis.

As expected, the number of trichomes decreases from young to mature leaves. Regarding the non-glandular trichomes, although their number is not much higher on the lower epidermis compared to the upper one, the degree of coverage is much higher on the lower epidermis (Figure 3D,F). This fact is due to their morphology—the trichomes on the upper epidermis are weakly branched, with short and few rays, or even unbranched, while those on the lower epidermis are massive, with numerous rays (10–16), and often layered in two or three circles.

### 2.2. Microscopic Description of Trichomes

The trichomes (glandular and non-glandular) from the leaves of *P. herba-venti* L. subsp. *pungens* were observed under the light microscope and the scanning electron microscope (SEM), and the glandular ones were examined under the confocal microscope as well. 

Glandular trichomes are quite variable in shape and size and can be divided into 2 categories: capitate (C type) and dendroid (D type). In the case of capitate trichomes, we can distinguish two subtypes: subtype C1—short trichomes, consisting of a basal cell, a stalk cell and 1–2 secretory cells (Figure 3G); subtype C2—longer trichomes, with a uni- or bicellular stalk and four secretory cells (Figure 3H). In all cases, the secreted material is located between the cell wall of the secretory cells and the cuticle.

Dendroid glandular trichomes are rarely found in plants and represent a hybrid between tector dendroid trichomes and secretory trichomes. In the analyzed species, most dendroid trichomes lack the secretory part. Their morphological aspect is also highly variable: they can present from 1 or 2 lateral branches to 5–6 or more than 10 branches (Figure 3I–L,N,O). The secretory head consists of one–four cells. The terminal cell of the stalk trichome, located beneath the secretory head, exhibits a distinct structure. These cells, known as neck cells, are shorter compared to the other cells in the stalk and possess unique properties.

Non-glandular trichomes are usually multicellular and branched (unicellular trichomes can be observed on the upper epidermis, even on fully expanded leaves). Those on the adaxial face are less branched, with a few short branches usually arranged in a single whorl. The trichomes on the abaxial side are massive, strongly branched, with the rays frequently arranged in two-tiered rings. For this reason, the degree of coverage is almost 100% at the level of the lower epidermis in the leaves at different stages of ontogenetic development (less at the level of the veins, where they are less dense). The surface of the branches of dendroid trichomes (both glandular and non-glandular) is covered with numerous micropapillae. 

Hydathodes, bordered by unicellular non-glandular trichomes, with two–three aqueous pores, are visible at the tip of the marginal teeth of the leaf (Figure 3P).

### 2.3. Histochemistry of Glandular Trichomes

In the control (unstained samples), the C1 and C2 capitate trichomes appear colorless. In the case of C1 trichomes, the secretion product can be observed in very small quantities, and only in some trichomes. However, the majority of C1 trichomes accumulate secretory products in glandular cells. This phenomenon was previously described in capitate trichomes from Lamiaceae species [18,22,23]. On the other hand, C2 type capitate trichomes, when mature, contain a significant amount of secretory product accumulated between the glandular cells and the cuticle. The secreted substance appears colorless and bright under normal light.

Glandular dendroid trichomes (D) also appear colorless, and the secreted product has a pale yellow color. This same color can be observed at the level of the neck cell.

The data obtained from the histochemical analyses indicate that the material secreted by the three types of trichomes has a complex chemical structure. The results of the histochemical tests for capitate and dendroid glandular trichomes are presented in Table 2. 

**Figure 4:**
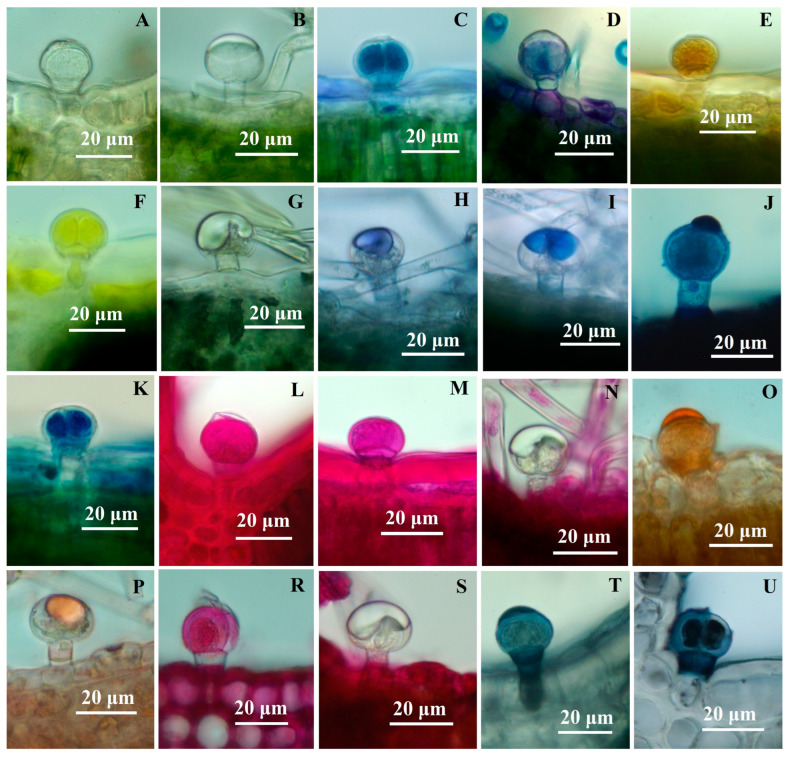
Light micrographs showing reaction to histochemical tests of capitate trichomes (**C**) to histochemical tests: (**A**) capitate trichome (C1), control; (**B**) capitate trichome (C2), control; (**C**)—capitate trichome (C1); toluidine blue; (**D**) apitate trichome (C2), toluidine blue; (**E**) C1, potassium dichromate; (**F**) C1, concentrated sulfuric acid; (**G**) C2, concentrated sulfuric acid; (**H**,**I**) C2, NADI reagent; (**J**) C2, Nile Blue; (**K**) C1, Nile Blue; (**L**,**M**) C1, PAS reagent; (**N**) C2, PAS reagent; (**O**) C1, Sudan Red; (**P**) C2, Sudan Red; (**R**) C1, Ruthenium Red; (**S**) C2, Ruthenium Red; (**T**,**U**) C1, Sudan Black.

**Figure 5:**
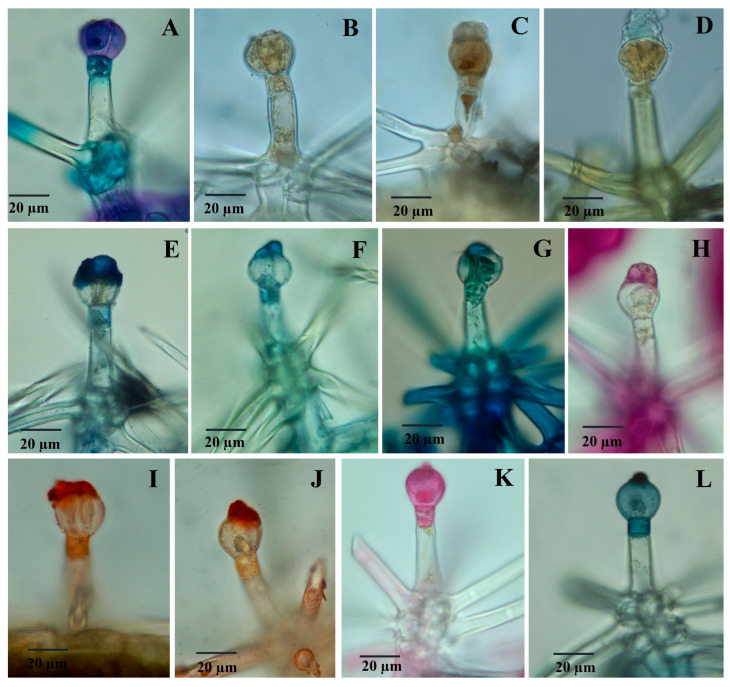
Light micrographs showing reaction to histochemical tests of dendroid trichomes (D) to histochemical tests: (**A**) toluidine blue; (**B**) potassium dichromate; (**C**) ferric chloride; (**D**) concentrated sulfuric acid; (**E**,**F**) NADI reagent; (**G**) Nile Blue; (**H**) PAS reagent; (**I**,**J**) Sudan Red; (**K**) Ruthenium Red; (**L**) Sudan Black.

Glandular trichomes present a mixture of both hydrophilic and lipophilic secretions. The secretion products differ in their chemical nature among the three types of glandular trichomes analyzed. Phenolic compounds were identified in the glandular cells, showing a strongly positive reaction in the case of capitate C1 (Figure 4C) and dendroid trichomes (Figure 5A). However, when tested for polyphenols, the secretion product from C2 capitate trichomes consistently showed a negative result (Figure 4D). 

Capitate glandular trichomes of C1-type exhibit a strong yellow color reaction when treated with concentrated sulfuric acid (positive for sesquiterpenes) (Figure 4F). On the other hand, C2-type glandular trichomes show a negative reaction both in the glandular cells and the secretion product (Figure 4G). Dendroid glandular trichomes show a positive reaction at the secretion level (Figure 5D).

The secretion product accumulated in the subcuticular space (for capitate trichomes C2 type) or excreted (for dendroid trichomes) is stained in blue or blue-violet with the NADI reagent (Figure 4H,I), which indicates the presence of volatile oils.

Polysaccharides are present (in a small amount) in the secretion product from dendroid trichomes, thus staining light red with the PAS reagent (Figure 5H), but they are absent from capitate trichomes C2 type (Figure 4N); C1-type glandular trichomes show positive reactions to the PAS reagent at the level of secretory cells (and negative for secretion products).

When stained with Ruthenium red, the dendroid trichomes showed a positive reaction at the level of the secretory cells and at the level of the neck cell (Figure 5K); capitate trichomes show a similar reaction to the PAS reagent to Ruthenium red staining (C2 type trichomes are constantly negative, both at the level of the secretory cells and the secretory product) (Figure 4S). Both capitate (Figure 4T,U) and dendroid trichomes (Figure 5L) show intense colors (red-orange) of secretion products when stained with Sudan III and dark-blue with Sudan Black, which indicates the presence of lipids. The positive reaction is also observed at the neck cell level from the dendroid trichomes.

### 2.4. Confocal Microscopy

**Figure 6:**
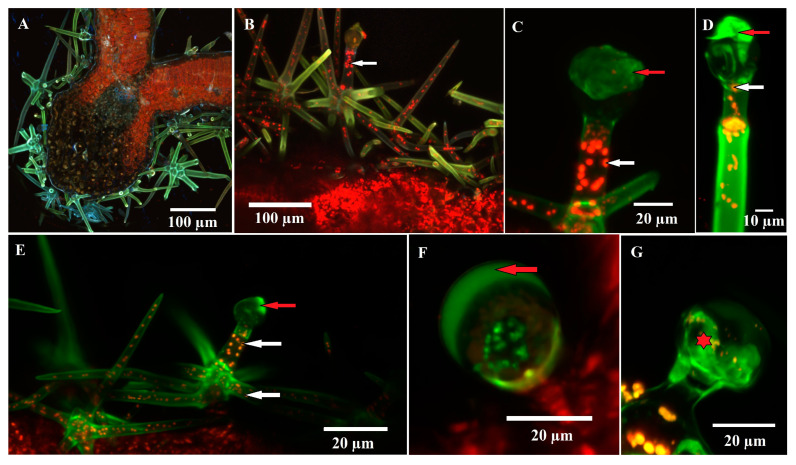
Confocal micrographs of glandular trichomes—autofluorescence of secreted materials and chloroplasts from the trichome cells could be observed: (**A**) cross section through the leaf (intermediate size) in the area of the midrib; (**B**–**E**) glandular dendroid trichome; (**D**) autofluorescence (green color) can be observed at the level of the secretion product (red arrow), especially at the trichome in the postsecretory phase (**D**). Chloroplasts, intensely autofluorescent (red color), are present in stalk cells, neck cells and trichome branches (white arrow); rare and small chloroplasts are visible even in the secretory cell; (**C**,**F**) capitate glandular trichome (C2); the secretion product is intensely autofluorescent; (**G**) capitate glandular trichome (C2); green color can be observed at the level of the secretory cells (star).

Confocal microscopy was used to observe natural autofluorescence in glandular trichomes. Chloroplasts, which are autofluorescent in red, were observed in large numbers in the assimilatory parenchyma (as expected) (Figure 6A), as well as in the cells of the dendroid glandular trichomes such as the stalk cells (and branches) (Figure 6B,C,E), the neck cell, and even a few small chloroplasts in the secretory cells (Figure 6C,D). The secretion product shows yellow-green autofluorescence in dendroid glandular trichomes and C2 capitate trichomes (Figure 6F). In the initial stages of development, autofluorescence can be observed at the level of the secretory cells (Figure 6G).

## 3. Discussion

The leaf indumentum is well developed in the *Phlomis verba-venti* subsp. *pungens* species. The presence of trichomes (especially glandular, but also non-glandular) is a characteristic feature of plants belonging to the Lamiaceae family [19,43]. The trichomes of the genus Phlomis, particularly those of *P. herba-venti*, exhibit distinct characteristics compared to those of other species in the same family. The trichomes from *P. herba venti* were described for the first time in detail (from a micromorphological and histochemical point of view) in this paper.

Glandular and non-glandular trichomes appear early in ontogenesis on both epidermises of the leaf lamina. The leaf indumentum of *P. herba-venti* covers the lower epidermis to a greater extent, where stomata are located. Non-glandular trichomes play a role in reducing transpiration, which is important for the species’ adaptation to relatively arid habitats. The species analyzed in this study was collected from a typical forest-steppe ecosystem, which receives approximately 550 mm/year of rainfall and experiences up to four months of drought during the summer [44].

A dense layer of non-glandular trichomes covering the epidermis is a xerophytic trait [45,46]. These trichomes exhibit great variability in the analyzed species, ranging from unicellular or weakly branched trichomes on the upper epidermis to strongly branched trichomes with 10–20 branches arranged in two–three tiers on the lower epidermis. Since unicellular trichomes are also found on the upper epidermis of mature leaves and strongly branched ones are found on the epidermis of very young leaves, we cannot consider these trichomes as being different stages of ontogenetic development. Variability in trichomes’ morphology has also been observed in other *Phlomis* species, such as *P. olivieri* [26] and *P. monocephala* [27], as well as in species of the genus *Phlomoides* [24]. The presence of micropapillae on the surface of the branches has a dual role: on the one hand, it enhances the hydrophobicity of the leaf surface, facilitating self-cleaning processes of the leaves [23,47]; on the other hand, it increases the resistance and thickness of the cell walls, thereby enhancing their protective role [48].

Glandular trichomes from *P. herba-venti* are of two categories: capitate and dendroid. The first category is frequently found in species from the Lamiaceae family, along with peltate trichomes which, although characteristic for the family, are missing in *P. herba-venti.*

Capitate trichomes (C1 and C2) are found on both epidermises, being more frequent on the upper epidermis, where they are predominantly located near the veins. Capitate trichomes have been observed in different species of the genus *Phlomis* [16,24,25] and numerous other species of the Lamiaceae family [13]. Similar to non-glandular trichomes, the number of glandular trichomes per surface unit decreases with leaf age. This phenomenon was also observed by other authors [3] and is due, on the one hand, to the increase in the surface of the leaf, but also to the lower protection requirements when they reach maturity.

Capitate trichomes in Lamiaceae-family species have a less consistent morphology than peltate ones [18] and can be used for taxonomic identification of plant species [14].

Glandular dendroid trichomes are a unique type of secretory and protective structures, which are rarely mentioned in the literature. They have been previously described in *Phlomis olivieri* [26], *P. monocephala* [27], *P. russeliana* (only on the corolla) [16], and *P. fruticosa* [25]. The dendroid glandular trichomes from *P. herba-venti* exhibit the same morphological variability as the non-glandular ones; trichomes with 1 non-glandular branch and 1 glandular one or with 2–16 non-glandular branches (arranged in tiers) can be observed. Moreover, we rarely observed trichomes with two terminal branches—one glandular and one non-glandular (Figure 2O). Apart from the species of *Phlomis*, in *Ballota undulata*, a branched biramous [49] glandular trichome was described (which has a single non-glandular branch, and is thus not a truly dendroid hair) [50].

Although there have been numerous investigations regarding the volatile oil components produced by the glandular trichomes on the leaves of *P. herba-venti*, these secretory structures have not yet been studied using histochemical techniques.

The analysis of volatile oils showed relatively large variations in chemical composition depending on the location where the plant material was collected. It was found that the predominant compounds were monoterpenes and sesquiterpenes. The presence of D-germacrene and α-pinene was consistently observed in all analyzed samples [28,29,30,31] (Table S1). 

It is well known that the chemical composition of volatile oils varies depending on the environmental conditions in which plants grow (soil, altitude, humidity, etc.), the time of collection, the ontogenetic stage, and the genetic structure of the studied population [29].

Several chemotypes have been identified in different species belonging to the genus *Phlomis* [51]; the variation in the components of the volatile oil from *P. herba-venti* suggests that these chemotypes are also found within the same species (intraspecifically), i.e., not only within the same genus (intragenerically).

Hexadecanoic acid (a saturated fatty acid in plants) was found to be an important constituent of the volatile oil from other species of the Lamiaceae family—*Scutellaria sp.* [52], *Satureja macrantha*, and *Nepeta betonicifolia* [53].

Histochemical analyses complement those of micromorphology and provide data on the secretion products of different categories of trichomes [54]. Glandular trichomes from *P. herba-venti* tested positive for most of the compounds analyzed using histochemical techniques. However, the localization of the various compounds is different; they are found either in the secretory cells, in the secretory products, or sometimes in both. In dendroid glandular trichomes, positive reactions were also sometimes identified in the neck cell in the case of tests for phenols, terpenes, and total lipids.

Capitate glandular trichomes of type C1 contain most of the secretory products at the glandular cell level (only a small amount can sometimes be observed in an extremely reduced subcuticular space). The presence of polyphenols, lipids, and polysaccharides in the glandular cells of this category of trichomes has also been described in other Lamiaceae species, such as *Marrubium vulgare* [18], *Ocimum obovatum* [22], and *Leucas lavandulaefolia* [23].

The secretion product in capitate trichomes, type C2, is located between the external wall of the secretory cells and the cuticle (subcuticular space). Frequently, the subcuticular localization of secretion products, especially volatile oil, is characteristic of peltate trichomes in species from the Lamiaceae family. However, in the case of the *P. herba-venti* species, peltate trichomes are absent. Maleci Bini and Giuliani [55] mention the case of rosemary, where the capitate trichomes (similar to those described in this paper as C2) produce volatile oil in a manner similar to the peltate ones, usually found in Lamiaceae species. 

The C1 type capitate trichomes do not produce detectable volatile oil in the subcuticular space, and the secretion of the dendroid trichomes is a mixture of polyphenols, lipids, and polysaccharides.

The presence of the 2 types of capitate trichomes identified in *P. herba-venti* was also observed in other species from the Lamiaceae family: *Ocimum obovatum* [22], *Nepeta congesta* [28], and *Plectranthus ornatus* [14]; they produce large amounts of volatile oils The volatile oil produced by C2 type capitate trichomes was identified by strong positive reactions when tested with NADI reagent and Sudan III. Polysaccharides are found in increased amounts in C1-type capitate trichomes, a fact proven by the positive reaction to PAS and Ruthenium Red staining. Phenolic compounds are found in smaller quantities in their secretory cells, along with sesquiterpenes and acidic lipids.

Dendroid glandular trichomes present a secretion product that is eliminated outside the secretory cells through the cuticle; it contains sesquiterpenes, phenolic compounds, polysaccharides, and lipids. In addition to the role of protection of young leaves (especially against herbivory and parasite attack) achieved through the intermediary of the glandular part, these trichomes also have a protective function, as observed in *P. fruticosa* by Nikolakaki and Christodoulakis [25]. This extra protection is particularly beneficial for species that have adapted to living in arid environments. 

The only paper that contains references to secretory trichomes from *P. herba-venti* is the paper by Özdemir [56], which deals with the anatomical peculiarities of the vegetative organs in two varieties of this species. In this context, only one category of capitate glandular trichomes is described, and the dendroid glandular trichomes are not shown. This can be explained by their presence predominantly on leaves that have not reached maturity, being rarer on fully expanded leaves, which may lead to their omission.

The neck cell (from the glandular dendroid trichomes) has a special structure, its walls being partially suberized, which ensures a controlled circulation of substances between the secretory head and stalk trichome; their role is to prevent the dissipation of the secretion product towards the base of the trichome [57].

The presence of chloroplasts (autofluorescence in red) [58] in stalk cells and in the branches of dendroid glandular trichomes was described for the first time; this confirms the fact that the trichomes are alive at maturity, although usually, non-glandular pluricellular trichomes (from which the ones analyzed by us derive) are dead at maturity (in order to fulfill their protective role more effectively); confocal microscopy investigations revealed the presence of chloroplasts in non-glandular trichomes as well. The presence of the functional protoplast in non-glandular trichomes was observed by Santos-Tozin [43] in Lamiaceae and Verbenaceae species.

The secretion product from the capitate trichomes (C2—accumulated in the subcuticular space) and from the dendroid glandular trichomes shows intense yellow-green autofluorescence; this is associated with the presence of flavonoids [59,60]. In *Solanum lycopersicum* and *S. habrochaites* trichomes, an intense yellow-green auto-fluorescence is apparent in the early stages of development, which disappears in the mature stages [59]. On the other hand, the presence of lipids was demonstrated by green autofluorescence in the secretory cells of trichomes from *Doronicum* species [60]. Yellow-green fluorescence was reported by Bergau et al. [61] to be a characteristic feature of glandular trichomes containing secretions [21].

The results obtained from the histochemical analyses confirm the presence of the compounds observed in the volatile oils analyzed by different authors but provide additional information regarding the distribution of different types of compounds within the glandular trichomes.

## 4. Materials and Methods

### 4.1. Plant Material

Leaves from *Phlomis herba-venti* subsp. *pungens* Maire ex DeFillips, in 3 different ontogenetic stages, were collected from the Valea lui David natural reservation (ROSCI0265), located close to the city of Iaşi (Romania) (47°11′31.5″ N, 27°28′06.8″ E and altitude 180 m). Collected leaves were sorted in 3 categories: very young leaf (4.73 ± 0.89 cm), intermediate leaf (6.76 ± 1.39 cm), and fully expanded leaf (11.59 ± 2.28 cm). A voucher specimen is stored in the Herbarium of Faculty of Biology, ”Alexandru Ioan Cuza” University of Iași, Romania (I-207020).

### 4.2. Light Microscopy

Small leaf pieces (from all 3 categories mentioned above) were fixed in glutaraldehyde (2% in 0.1M Phosphate Buffer) at 4 °C for 2 h, dehydrated in a graded ethanol series, and embedded in Epon 812 resin. Semithin sections were obtained using glass knifes and a Power-Tome PT-X ultramicrotome. The sections were placed in a drop of water, dried on a hot plate, and stained with toluidine blue (0.05% aqueous solution). Photographs were taken with an Olympus E-330 photo camera, using an Olympus BX41 research microscope. Measurements (n = 10) for the height of the palisade parenchyma and spongy parenchyma were made using IC Measure Imaging Source software.

### 4.3. Scanning Electron Microscopy (SEM)

Fragments of leaves at different developmental stages were fixed in glutaraldehyde (2.5%) for 4 h and stored in 70% ethanol (Johansen 1940). After dehydration in a graded ethanol series and acetone (100%, twice), the vegetal material was dried with CO_2_ in an EMS 850 critical point dryer, mounted on stubs with carbon conductive tape, sputter-coated with a 30 nm layer of gold (EMS 550X Sputter Coater), and examined by scanning electron microscope (Tescan Vega II SBH) (from the Electron Microscopy Laboratory, Faculty of Biology, Alexandru Ioan Cuza University of Iași). 

The density of glandular and non-glandular trichomes was measured on micrographs obtained by SEM; the trichomes of a certain category were counted on a surface unit of known size. The measurements were made using IC Measure Imaging Source software. If the trichome was over 50% in the analyzed area, it was counted with the value 1; if less than 50% of the trichome was in the analyzed surface, it was counted with the value 0. The obtained values represent the average (n = 10) ± standard deviation.

### 4.4. Histochemical Investigations

Free hand sections made on fresh material were subjected to histochemistry analyses; the following reactions were carried out: toluidine blue for phenols [33], potassium dichromate [35] and ferric chloride for phenolic compounds [34], concentrated sulfuric acid for sesquiterpenes [36], NADI reagent for terpenes/essential oils [37], Nile Blue for neutral and acidic lipids [38], PAS reagent for polysaccharides [39], Sudan Red for total lipids [40], Ruthenium Red for acid polysaccharides (other than cellulose) [41], and Sudan black for total lipids [42].

### 4.5. Confocal Microscopy

A Nikon AX R Eclipse Ti2-E Confocal Microscope system equipped with a motorized inverted optical support model (Ti2-E) and a Galvano scanner with a CFI Plan Apochromat Lambda D optical system and a LUA-S6 laser unit with 6 wavelengths—405, 445, 488, 514, 561, and 640—was used for sample visualization (free hand sections were made on fresh material); the images acquisition and analysis were made with the software, Nis Elements Ai (Artificial Intelligence for automatically removing blur from widefield fluorescence microscope images).

The laser wavelengths used for excitation in the analysis of the samples in this paper were 405, 488, 561, and 640. Access to the research equipment was offered by ELTA’90 Medical Research SRL.

## Figures and Tables

**Table 1:** Density of glandular and non-glandular trichomes in leaves in different stages of development.

Developmental Stage of the Leaf	Non-Glandular Trichomes/mm^2^		Glandular Capitate Trichomes/mm^2^		Glandular Dendroid Trichomes/mm^2^	
	Upper Epidermis	Lower Epidermis	Upper Epidermis	Lower Epidermis	Upper Epidermis	Lower Epidermis
Very young leaf (S1)	222.17 ± 44.71	225.70 ± 37.38	120.46 ± 31.57	56.12 ± 17.44	0	8.54 ± 8.23
Intermediate leaf (S2)	68.91 ± 9.15	95.82 ± 44.51	41.97 ± 15.34	25.39 ± 19.55	1.55 ± 2.5	14.36 ± 16.24
Fully expanded leaf (S3)	62.57 ± 9.28	86.94 ± 11.83	34.89 ± 15.84	17.38 ± 9.16	1.06 ± 3.35	2.89 ± 6.1

Data represents the density of the trichomes—number per mm^2^ of leaf epidermis (average ± standard error).

**Table 2:** Histochemical identification of compounds from glandular trichomes (capitates: C1 and C2; and dendroids: D) from *Phlomis herba venti* leaf.

Staining Technique	Target Product/Authors	Glandular Capitate Trichomes (C1)		Glandular Capitate Trichomes (C2)		Glandular Dendroid Trichomes (D)	
		Color	Figure	Color	Figure	Color	Figure
Toluidine Blue	Phenols (Gutmann 1995) [33]	Blue	4C	Violet blue	4D	Blue	5A
Potassium dichromate	Phenolic compounds (Gabe, 1968) [34]	None	Not shown	Brown	4E	Light brown	5B
Ferric chloride	phenolic compounds (Gahan, 1984) [35]	Brown	Not shown	Brown	Not shown	Brown	5C
Concentrated sulfuric acid	sesquiterpenes (Cappellatti et al., 1986) [36]	Yellow	4F	No color	4G	Dark yellow	5D
NADI reagent	terpenes /essential oil (David and Carde, 1964) [37]	None	Not shown	Blue-violet	4H, I	Blue	5E, F
Nile Blue	Neutral lipids (pink) and acid lipids (blue) (High, 1984) [38]	Blue	4K	Blue	4J	Blue	5G
PAS reagent	Polysaccharides (McManus, 1948) [39]	Red	4L, M	None	4N	Light red	5H
Sudan Red	Total lipids (Brundett el al., 1991) [40]	Orange—red	4O	Orange—red	4P	Orange—red	5I, J
Ruthenium Red	Acid polysaccharides (Johansen, 1940) [41]	Pink	4R	No color	4S	Pink	5K
Sudan black	Total lipids (Pearse, 1985) [42]	Dark blue	4T, U	Dark blue	Not shown	Dark blue	5L

## Data Availability

All data are available in the supplement or upon request.

## Supplementary Materials

The following supporting information can be downloaded at: https://www.mdpi.com/article/10.3390/plants12132423/s1, Table S1 The main constituents of volatile oils in *Phlomis herba* venti with different geographical origins (identified by GC and GC-MS analyses).
