# Supplementary material for: Glandular and Non-Glandular Trichomes from Phlomis herba-venti subsp. pungens Leaves: Light, Confocal, and Scanning Electron Microscopy and Histochemistry of the Secretory Products
Source: Plants (Basel). 2023 Jun 23;12(13):2423. doi: 10.3390/plants12132423 (PMC10347251; doi:10.3390/plants12132423)
Supplement: Supplementary file 1 [file plants-12-02423-s001.zip › plants-2408322-supplementary.pdf]

Table S1. The main constituents of volatile oils in *Phlomis herba venti* with different geographical origins (identified by GC and GC-MS analyses).

Chemical family	Components	Origin	Reference
Sesquiterpens	Germacrene D -11.7%, β-bourbonene – 7.3%, β –caryophyllene – 5.0%	Shanjan Region, Iran	Delnavazi et al, 2014 [28]
	Germacrene D -31.1%, T-muurolol – 11.0%, β –caryophyllene – 1.7%, β-bourbonene – 1.5%	Mazandaran province, northern Iran	Khalilzadeh et al, 2008 [30]
	Germacrene D -7.2%, β-bourbonene – 0.8%, β –caryophyllene – 0.6%	Ankara, Turkey	Sarikurkcu et al, 2016 [29]
	Germacrene D -33.9%, α-muurolene – 4.2%, β-bourbonene – 4.0%, β –caryophyllene – 2.4%	Mazandaran province, northern Iran	Morteza-Semnani et al, 2004 [32]
	Germacrene D -24.5%, β-farnesene – 13.4%, bicyclogermacrene – 14.1%	Orromieyeh, Province, Iran	Masoudi et al, 2006 [31]
Monoterpenes	α-pinene – 7.3%, terpinolene – 9.1%	Shanjan Region, Iran	Delnavazi et al, 2014 [28]
	α-pinene – 7.1%	Mazandaran province, northern Iran	Khalilzadeh et al, 2008 [30]
	α-pinene – 13.5%	Ankara, Turkey	Sarikurkcu et al, 2016 [29]
	α-pinene – 9.4%	Mazandaran province, northern Iran	Morteza-Semnani et al, 2004 [32]
	α-pinene – 13.5%	Orromieyeh, Province, Iran	Masoudi et al, 2006 [31]
Hexadecanoic acid (palmitic acid)	7.4%	Shanjan Region, Iran	Delnavazi et al, 2014 [28]
	-	Mazandaran province, northern Iran	Khalilzadeh et al, 2008 [30]
	68.1%	Ankara, Turkey	Sarikurkcu et al, 2016 [29]
	12.9%	Mazandaran province, northern Iran	Morteza-Semnani et al, 2004 [2004]
	0.1%	Orromieyeh, Province, Iran	Masoudi et. al, 2006 [31]
